# The Moderating Effect of Attention-Deficit Hyperactivity Disorder Symptoms on the Relationship Between Procrastination and Internalizing Symptoms in the General Adult Population

**DOI:** 10.3389/fpsyg.2021.708579

**Published:** 2021-10-26

**Authors:** Mana Oguchi, Toru Takahashi, Yusuke Nitta, Hiroaki Kumano

**Affiliations:** ^1^Graduate School of Human Sciences, Waseda University, Tokorozawa, Japan; ^2^Japan Society for the Promotion of Science, Tokyo, Japan; ^3^Faculty of Human Sciences, Waseda University, Tokorozawa, Japan

**Keywords:** attention-deficit hyperactivity disorder, procrastination, depression, anxiety, emerging adulthood

## Abstract

**Background:** The symptoms of attention-deficit hyperactivity disorder (ADHD) are known to exacerbate the effect of cognitive-behavioral impairments on emotional burden. Although adults with ADHD frequently experience procrastination and internalizing symptoms such as depression and anxiety, few studies have examined whether the association between procrastination and internalizing symptoms differs by ADHD symptoms.

**Objective:** This study aimed to examine the moderating effect of ADHD symptoms on the association between procrastination and internalizing symptoms.

**Method:** A cross-sectional survey was conducted among 470 adults (mean age=26.57, standard deviation=2.93) using self-reported questionnaires: Adult ADHD Self-Report Scale, General Procrastination Scales, Patient Health Questionnaire-9, and State–Trait Anxiety Inventory.

**Conclusion:** Participants with more substantial ADHD symptoms experienced more procrastination and internalizing symptoms than those with the less substantial ADHD symptoms. Therefore, procrastination constitutes the treatment target for those suffering from ADHD and comorbid internalizing symptoms. Alternatively, there was no enhancing effect of ADHD symptoms on the association between procrastination and internalizing symptoms. It is necessary to examine more precise and valid hypotheses and underlying mechanisms of procrastination in high and low ADHD symptom groups.

## Introduction

Attention-deficit hyperactivity disorder (ADHD), which persists in 2.5–5% of adults (McCarthy et al., 2012; Polanczyk et al., 2014), is characterized by inattention and hyperactivity/impulsivity (American Psychiatric Association, 2013). People diagnosed with ADHD are likely to suffer from comorbid emotional conditions such as depression and anxiety (Chen et al., 2018). Severe depressive symptoms are commonly experienced by adults (Kessler et al., 2006; Torgersen et al., 2006) and emerging adults (aged 18–24years; Arnett et al., 2014) with ADHD (Biederman et al., 2010; Hinshaw et al., 2012; Meinzer et al., 2013). Adults with ADHD are also at a higher risk for developing anxiety disorders than the general population (Kessler et al., 2006). These emotional symptoms, usually summarized as “internalizing symptoms” (McElroy and Patalay, 2019), affect the life satisfaction of adults with ADHD (Yang et al., 2013).

To improve internalizing symptoms, it is necessary to approach the associated symptoms of ADHD as well as decreasing the cardinal symptoms (Safren et al., 2004, 2005; Knouse et al., 2013a). The associated symptoms occur because of a lack of skills for managing the cardinal symptoms of ADHD. Adult ADHD patients have such associated symptoms as procrastination, low frustration tolerance, severe mood swings, and low motivation, leading to various problems in daily life (Weiss and Weiss, 2004). Among these associated symptoms, adult ADHD patients experience procrastination frequently in their daily lives (Young and Bramham, 2007; Ramsay, 2017). Procrastination has been categorized as one of the functional disorders associated with the inattention symptom cluster of ADHD (Young and Bramham, 2007). According to the DSM-5 criteria, inattentive symptoms are associated with procrastination: “often does not follow through on instructions and fails to finish schoolwork, chores, or duties in the workplace (e.g., loses focus, side-tracked)”; “often has trouble organizing tasks and activities”; and “often avoids, dislikes, or is reluctant to do tasks that require mental effort over a long period of time (such as schoolwork or homework).” In a cross-sectional study of college students without an ADHD diagnosis, only inattentive symptoms were positively associated with procrastination after controlling for impulsivity/hyperactivity, while the correlation between impulsivity/hyperactivity and procrastination disappeared by controlling for inattention as measured by a questionnaire (Niermann and Scheres, 2014).

Procrastination has two aspects: maladaptive and adaptive. Maladaptive procrastination is defined as “voluntarily delay in an intended course of action despite expecting to be worse off due to the delay” (Steel, 2007). Several studies have found that procrastination is associated with high levels of depression and anxiety symptoms among emerging adults (van Eerde, 2003; Beutel et al., 2016). This maladaptive behavior also causes low work efficiency, financial difficulties, and poor academic achievement (Stead et al., 2010). In the general population, procrastination not only has maladaptive behaviors, but also adaptive aspects. The positive forms of delay (e.g., active procrastination; Chu and Choi, 2005) refer to self-managed time-management strategy for working under pressure and successfully meeting deadlines. The common element for both aspects of procrastination is the intentional delay, which is a personally important action (Klingsieck, 2013). Alternatively, the difference between both concepts is that maladaptive procrastination includes unnecessary and unreasonable delay (Steel, 2010). For example, college students who showed more adaptive procrastination had higher grades (Choi and Moran, 2009; Kim et al., 2017), and adaptive procrastination was positively correlated with intrinsic motivation, well-being (Habelrih and Hicks, 2015), self-efficacy, and self-regulation (Taura et al., 2015). In contrast to maladaptive procrastination, adaptive procrastination has positive psychosocial consequences due to the delay.

Procrastination, which can cause maladaptive problems, frequently appears in ADHD adults’ daily lives (Ramsay, 2020). Clinically, adults with ADHD experience procrastination as the most common functional impairment (Solanto et al., 2010; Ramsay, 2020). Chronic procrastination occurred more frequently in ADHD patients than those without ADHD regardless of gender (Ferrari and Sanders, 2006). Those with chronic procrastination were particularly prone to procrastination with thrill-seeking and avoidance functions. Furthermore, adults with ADHD procrastinate as a maladaptive compensatory strategy to avoid negative or stressful situations when faced with them (Ramsay and Rostain, 2008). Therefore, adults with ADHD might have more maladaptive procrastination compared to those without. These avoidant behaviors are major maintenance and aggravation factors common to depressive and anxiety symptoms (Ehrenreich-May and Chu, 2013). Similarly, in adult ADHD, avoidance behavior leads to internalizing symptoms (Bodalski et al., 2019). Previous studies have suggested that adults with ADHD might experience procrastination as more maladaptive and show a stronger relationship between procrastination and internalizing symptoms than healthy controls. However, principally, the relationship between procrastination and internalizing symptoms has only been studied in the general population, and little is known in the clinical population, such as people with ADHD.

The presence of ADHD tendencies may explain the inconsistent research on the effects of procrastination on psychiatric symptoms. These inconsistent findings show that effects of procrastination differ depending on the presence of ADHD tendencies. For instance, the adaptive aspects of procrastination require executive functional skills such as time management and planning strategies (van Eerde et al., 2015). Additionally, strategic putting off also demands skills related to reward sensitivity, assessing the consequences of behavior in terms of their long-term advantage or disadvantage, and engaging in or waiting to acquire the long-term advantages and avoid the disadvantages (Safren et al., 2004; Rozental and Carlbring, 2014). However, since ADHD symptoms display deficits in executive function and delayed reward aversion (Sonuga-Barke et al., 2010), these impairments prevent the skill of putting off adaptively. In addition to children, adult ADHD patients have diminished performance on executive function tasks, such as response inhibition and planning/organization, compared to the control group (Fabio and Caprì, 2017; Martino et al., 2017). Additionally, children with ADHD indicate difficulty waiting for delayed rewards and tendencies to choose immediate rewards when memory load is heavier (Fabio et al., 2020). Those adult ADHD patients with more executive dysfunction and reward impairments tend to display the greatest impairment in daily life functioning. Therefore, adaptive procrastination is reduced in cases with high ADHD tendencies, which may exacerbate the relationship between procrastination and clinical symptoms such as depression and anxiety. In the clinical practice, adult ADHD patients have been reported to experience more of maladaptive procrastination (Ramsay and Rostain, 2008). Furthermore, people with ADHD in emerging adulthood struggle with demands for autonomy and self-regulation, which overwhelm their cognitive capacities (Knouse and Fleming, 2016). Nonetheless, the synergistic effects of ADHD and procrastination on internalizing symptoms remain unclear. Procrastination is one of the most prevalent difficulties faced by adults with ADHD and may further exacerbate their internalizing symptoms.

Therefore, the current study aimed to investigate the moderating effect of ADHD symptoms on the association between procrastination and internalizing symptoms. Based on the literature regarding ADHD, procrastination, and internalizing symptoms, this study proposed the following three hypotheses: (a) People with ADHD exhibit more procrastination, (b) procrastination is positively correlated with depression and anxiety symptoms, and (c) ADHD symptoms strengthen this correlation.

## Materials and Methods

### Ethical Considerations

All procedures of this study were approved by the institutional Ethics Review Committee on Research with Human Subjects of the first author’s affiliation (2018-282).

### Participants

The online questionnaire survey was conducted among participants who had registered on an online survey company in Japan. It was executed during February and March 2019. Before completing the questionnaire, participants read an onscreen explanation about responding without compulsion as an ethical consideration. This study recruited 500 participants who were grouped according to their Adult ADHD Self-Report Scale-v1.1 (ASRS) score. The higher-level ADHD symptoms group (ADHD-H) were those who scored above the cutoff point of the ASRS Part A (Kessler et al., 2005), while the lower-level ADHD symptoms group (ADHD-L) included those with scores below the cutoff point; both groups had 250 participants each. The sample size was calculated using G power 3.1.9.4 with 0.01 alpha and 0.95 statistical power. The effect size was determined using recent reports on the correlation between the status of ADHD symptoms and procrastination (*r*=0.21, Ozel-Kizil et al., 2016; *r*=0.43, Niermann and Scheres, 2014) and between procrastination and depression/anxiety (*r*=0.36, Beutel et al., 2016; *r*=0.27, Hernández et al., 2019). The average effect size was *r*=0.25, which resulted in an estimated sample size of 240. As a reward, respondents received points that could redeem for goods within the system of the survey company.

To ensure the quality of the online survey, data from 10 of the 500 participants were excluded because their answers showed patterns of straightlining for all scales. Straightlining is defined as giving the same answer for all items in each scale (Simon, 1956; Schonlau and Toepoel, 2015); it is a type of satisficing response that has been used as an indicator of poor answer quality (Zhang and Conrad, 2013). Of the five measures in the current study, 339 (67.8%) participants did not show straightlining in any measure. Further, 88 (17.6%) participants showed straightlining in at least one measure, 36 (7.2%) in two, 17 (3.4%) in three, and 10 (2%) in four. The remaining 10 participants with straightlining in all five measures were excluded from the analyses for their refusal to answer. The final sample consisted of 490 participants (244 men; age: mean=26.56, standard deviation=2.91).

### Measurements

#### ADHD Symptom Status

Attention-deficit hyperactivity disorder symptom status was assessed using the ASRS (Kessler et al., 2005). The first six items of this scale (Part A) are used for brief screening of ADHD symptoms in people aged 18years or older. This scale was scored using a 5-point Likert scale from 0 (*never*) to 4 (*very often*) to indicate ADHD symptoms. Responses like “*often*” and “*very often*” in all items were regarded as positive ADHD symptomatology. In the case of items 1, 2, and 3, a response of “*sometimes*” was also considered positive. This study defined ADHD-H as those who showed four or more positive responses for the first six items of the ASRS (Part A), which predicts an adult ADHD diagnosis based on the DSM-IV criteria (Kessler et al., 2007). This scale has high validity regarding the clinical diagnosis of ADHD as well as internal consistency reliability (Cronbach’s *α*=0.63–0.72) and test–retest reliability (*r*=0.58–0.77), and the area under the receiver operating characteristics curve (AUC) was 0.90 (Kessler et al., 2007).

#### Procrastination

Self-reported difficulties related to procrastination were determined using the General Procrastination Scale (GPS; Lay, 1986). The original English version of the GPS consists of 20 items, scored using a 5-point Likert scale from 1 (*extremely uncharacteristic*) to 5 (*very characteristic*). However, the Japanese version of the GPS (J-GPS; Hayashi, 2007) includes only 13 items, since seven were excluded during the translation and validation process. Higher scores indicate greater difficulties in everyday tasks, for example, “I often find myself performing tasks that I had intended to do the day before.” The Japanese version of the GPS has good construct validity and internal consistency reliability (Cronbach’s *α*=0.87) according to Hayashi (2007).

#### Depression

The Patient Health Questionnaire (PHQ-9; Kroenke et al., 2001) consists of nine items, scored using a 4-point Likert scale from 0 (*not at all*) to 3 (*nearly every day*). Higher scores indicate greater severity of depressive symptoms over the past 2weeks. The total score ranges from 0 to 27; scores over 20 indicate clinical severity. Internal consistency of the PHQ-9 has been shown to be high (Cronbach’s *α*=0.89; Kroenke et al., 2001, 2010). The reliability and validity of the Japanese version of the PHQ-9 were confirmed by Muramatsu et al. (2007).

#### Trait Anxiety

The Japanese version of the State–Trait Anxiety Inventory (Hidano et al., 2000) is scored using a 4-point Likert scale from 1 (*almost never*) to 4 (*almost always*). The inventory consists of two subscales (state and trait anxiety) with 20 items each. However, this study used only the trait anxiety items to investigate the frequency anxiety which a person experiences anxiety with the highest stability over time. Higher scores indicate greater severity of anxiety symptoms. The total score ranges from 20 to 80, and the clinical significance level is over 55. The reliability and validity of the Japanese version of the State–Trait Anxiety Inventory were confirmed by Hidano et al. (2000): internal consistency reliability (Cronbach’s *α*=0.896 in men and *α*=0.904 in women) and test–retest reliability (*r*=0.58 in men and *r*=0.77 in women).

### Statistical Analyses

All statistical analyses were conducted using IBM SPSS Statistics 26.0 software (SPSS Inc., 2017). The analyses included the following four steps. First, descriptive statistics were calculated to check for data normality where skewness within −1.00 to +1.00 indicated a normal distribution. Second, each scale’s scores were compared between groups by *t* tests to examine the first hypothesis. Third, Pearson’s correlation coefficients were calculated between all variables to test the second hypothesis. Finally, two hierarchical multiple regression analyses using depression and anxiety symptoms as outcome variables were conducted to examine the last hypothesis. Four variables (gender, age, ADHD symptom status, and procrastination) were used as predictors in the first step. In the second step, the interaction variable (ADHD symptom status and procrastination) was added. The interaction term was calculated using ADHD symptom status and the centering procrastination score. It aimed to establish the moderating effect of ASRS Part A on the association between procrastination and symptoms of depression and anxiety. The model fit was assessed using the Akaike information criterion (AIC) and Bayesian information criterion (BIC).

## Results

### Descriptive Characteristics

An acceptable level of skewness was observed for all scales, indicating a normal distribution. The descriptive characteristics and *t* test for all variables are presented in Table 1 including mean, standard deviation, kurtosis, skewness, Cronbach’s alpha, *t* ratio, value of *p*, and Cohen’s *d*. The number of participants with clinical-level depression and anxiety in the ADHD-H group was 24 (9.68%) and 90 (36.29%), respectively, and 13 (5.37%) and 43 (17.77%), in the ADHD-L group, respectively.

**Table 1:** Sample characteristics and descriptive statistics for all analytic variables.

	ADHD-H (*n*=248)		ADHD-L (*n*=242)		*t*	*p*	Cohen’s *d*	Kurtosis	Skewness	Cronbach’s *α*
	Mean	*SD*	Mean	*SD*						
Age	26.35	3.13	26.77	2.66	−1.61	0.108	0.14	−0.16	−0.76	
ASRS Part A	15.04	2.74	8.19	3.45	24.29	0.000	2.20	0.31	−0.28	0.83
GPS	42.27	7.97	35.49	8.40	9.17	0.000	0.83	0.21	−0.13	0.84
PHQ	9.40	7.01	6.52	6.55	4.69	0.000	0.42	−0.12	0.81	0.92
STAI-T	53.26	9.99	47.57	9.57	6.43	0.000	0.58	0.67	0.06	0.88

ASRS Part A, Adult ADHD Self-Report Scale-v1.1 Part A; GPS, General Procrastination Scale; PHQ, Patient Health Questionnaire-9; SD, standard deviation; STAI, State–Trait Anxiety Inventory-Form JYZ; ADHD-H, participants who scored above the ASRS Part A cutoff point; and ADHD-L, those who scored below the ASRS Part A cutoff point. Degrees of freedom=488.

### Comparing Levels of Procrastination and Internalizing Symptoms Between the ADHD-H and ADHD-L Groups

Our analyses revealed significant differences between the two groups for all assessments (Table 1). Participants with more ADHD symptoms reported higher levels of procrastination, depression, and anxiety compared to those with fewer ADHD symptoms.

Table 2 shows the correlations among total ASRS Part A score, procrastination, depression, and anxiety. Pearson’s correlations revealed significantly positive correlations among procrastination and depression and anxiety.

**Table 2:** Correlations among all variables.

	2.	3.	4.
1. ASRS Part A	0.42^***^	0.28^**^	0.35^***^
2. GPS		0.28^***^	0.39^***^
3. PHQ-9			0.60^***^
4. STAI-T			

*N*=490; ASRS Part A, Adult ADHD Self-Report Scale-v1.1 Part A; GPS, General Procrastination Scale; PHQ, Patient Health Questionnaire-9; and STAI, State–Trait Anxiety Inventory-Form JYZ.

** *p*<0.01,

*** *p*<0.001.

### Moderation of the Association Between Procrastination and Internalizing Symptoms by ADHD Symptom Status

Hierarchical multiple regression analyses using depressive and anxiety symptoms as outcome variables were conducted using all variables as predictors in the first step and adding the interaction of ADHD symptom status and procrastination as a predictor in the second step. These analyses allowed the examination of whether procrastination predicted internalizing symptom and if this relation is strengthened by ADHD symptom status as a moderator.

The results for depressive symptoms are presented in Table 3 and for anxiety symptoms are presented in Table 4. These analyses revealed that, in the first step, both ADHD symptom status [*β*=−0.121, *B*=−1.675, *SE*=0.651, *p*=0.010, CI (−2.953, −0.397)] and procrastination [*β*=0.232, *B*=0.182, *SE*=0.037, *p*<0.001, CI (0.109, 0.254)] had a statistically significant effect on depression after controlling for participants’ sex and age. ADHD symptom status [*β*=−0.152, *B*=−3.085, *SE*=0.909, *p*=0.001, CI (−4.871, −1.300)] and procrastination [*β*=0.341, *B*=0.392, *SE*=0.052, *p*<0.001, CI (0.290, 0.493)] also had a significant effect on trait anxiety. In contrast, in the second step, the interaction effect of ADHD symptom status and procrastination was not significantly associated with depression [*β*=−0.010, *B*=−0.017, *SE*=0.074, *p*=0.813, CI (−0.162, 0.128)] or trait anxiety [*β*=0.004, *B*=0.010, *SE*=0.103, *p*=0.919, CI (−0.192, 0.213)]. This interaction did not improve the prediction of depression (Δ*R*^2^<0.000, *p*=0.813, AIC=1862.159, BIC=1887.325) and anxiety symptoms (Δ*R*^2^<0.000, *p*=0.919, AIC=2189.647, BIC=2214.813) compared to first step in depression (AIC=1860.215, BIC=1881.187) and anxiety symptoms (AIC=2187.657, BIC=2208.629).

**Table 3:** Attention-deficit hyperactivity disorder symptom status, procrastination, and their interactions with PHQ.

	*β*	*B*	*p*	95% CI		*R* ^2^	Adjusted *R*^2^	*ΔR* ^2^	*F*	*p*	AIC	BIC
				Lower	Upper							
Step 1						0.090	0.083		11.997	0.000	1,860.215	1,881.187
Sex	0.017	0.229	0.704	−0.956	1.414							
Age	0.028	0.067	0.516	−0.137	0.271							
ADHD symptom status	−0.121	−1.675	0.010	−2.953	−0.397							
GPS	0.232	0.182	<0.001	0.109	0.254							
Step 2						0.090	0.081	0.000	9.590	0.000	1,862.159	1,887.325
Sex	0.016	0.222	0.714	−0.966	1.410							
Age	0.029	0.069	0.508	−0.136	0.274							
ADHD symptom status	−0.121	−1.673	0.010	−2.953	−0.394							
GPS	0.233	0.182	<0.001	0.109	0.255							
ADHD symptom status × GPS	−0.010	−0.017	0.813	−0.162	0.128							

*N*=490; Sex: male=1, female=2; ADHD symptoms status, four or more positive responses=1, less than four responses=2 in the Adult ADHD Self-Report Scale Part A; GPS, General Procrastination Scale; PHQ-9, Patient Health Questionnaire-9; *β*, standardized effect; *B*, unstandardized effect; 95% CI refers to unstandardized coefficient *B*; Δ*R*^2^, change of *R*^2^; AIC, Akaike information criterion; and BIC, Bayesian information criterion.

**Table 4:** Attention-deficit hyperactivity disorder symptom status, procrastination, and their interactions with STAI-T.

	*β*	*B*	*p*	95% CI		*R^2^*	Adjusted *R*^2^	Δ*R*^2^	*F*	*p*	AIC	BIC
				Lower	Upper							
Step 1						0.176	0.169		25.941	0.000	2,187.657	2,208.629
Sex	−0.021	−0.418	0.620	−2.073	1.237							
Age	0.041	0.144	0.321	−0.141	0.429							
ADHD symptom status	−0.152	−3.085	0.001	−4.871	−1.300							
GPS	0.341	0.392	<0.001	0.290	0.493							
Step 2						0.176	0.168	0.000	20.713	0.000	2,189.647	2,214.813
Sex	−0.020	−0.413	0.625	−2.073	1.246							
Age	0.041	0.143	0.326	−0.143	0.429							
ADHD symptom status	−0.152	−3.086	0.001	−4.874	−1.299							
GPS	0.341	0.391	<0.001	0.290	0.493							
ADHD symptom status × GPS	0.004	0.010	0.919	−0.192	0.213							

*N*=490; Sex: male=1, female=2; ADHD symptoms status, four or more positive responses=1, less than four responses=2 in the Adult ADHD Self-Report Scale Part A; GPS, General Procrastination Scale; STAI, State–Trait Anxiety Inventory-Form JYZ; *β*, standardized effect; *B*, unstandardized effect; 95% CI refers to unstandardized coefficient *B*; Δ*R*^2^, change of *R*^2^; AIC, Akaike information criterion; and BIC, Bayesian information criterion.

## Discussion

The present study aimed to examine the moderating effects of ADHD symptom status on the association between procrastination and internalizing symptoms. First, our analysis confirmed that adults with a higher ADHD symptom status showed more frequent procrastination than those with a lower ADHD symptom status. Participants in the ADHD-H group had higher scores for procrastination than those in the ADHD-L group, indicating a non-negligible effect size of Cohen’s *d* (0.83). This result is consistent with a previous study that compared academic procrastination in undergraduate students with or without ADHD symptoms status (Cohen’s *d*=0.65; Wood et al., 2019). Furthermore, the results of this study regarding participants with nonclinical ADHD symptoms are in line with previous findings that adults with ADHD diagnosis exhibit more procrastination (Ramsay, 2020). This suggests that procrastination constitutes an essential treatment target even for adults who are not diagnosed with but exhibit subthreshold ADHD symptoms. Second, the present study further confirmed the association between procrastination and symptoms of depression and anxiety, as observed in earlier studies (Solomon and Rothblum, 1984; van Eerde, 2003). This study observed a positive correlation between procrastination and depression and anxiety. Procrastination causes impairments in several daily life domains (Stead et al., 2010), which may, in turn, lead to internalizing symptoms.

Third, contrary to our hypothesis, ADHD symptom status had no moderating effect – that is, the association between procrastination and internalizing symptoms did not depend on the severity of ADHD symptoms. Although clinical perspectives have indicated that adults with ADHD are more likely to experience maladaptive procrastination in their daily lives (Ramsay and Rostain, 2008; Ramsay, 2020), our result did not provide any positive evidence to support it. The present study revealed that maladaptive procrastination is not a categorical problem due to ADHD symptoms. This result, which focused on procrastination as a behavioral impairment, is inconsistent with an earlier finding suggesting that ADHD exacerbates the association between cognitive burden and stress responses (Hirvikoski et al., 2009) and job functioning and conduct problems in young ADHD (Babinski et al., 2017).

Although this study did not support the hypothesis, its findings can be regarded as a significant step in understanding the role of ADHD in the onset of the procrastination. The current result suggested that association between procrastination and internalizing symptoms was not moderated by ADHD symptomatology, inattention, hyperactivity, and impulsivity. However, among the ADHD core symptoms, inattentiveness was found to be positively associated with elevated neuroticism (Knouse et al., 2013b), which predicts more frequent *maladaptive* procrastinations (Kim et al., 2017). Contrastingly, hyperactivity is positively associated with higher extroversion (Knouse et al., 2013a), which predicts more frequent *adaptive* procrastinations (Kim et al., 2017). Different ADHD symptoms may be associated with different aspects of procrastination. The ASRS used in this study was developed for screening ADHD and is unsuitable for measuring levels of different ADHD symptoms. Future studies should include scales that measure the different core symptoms of ADHD.

### Limitations

The present study has several limitations. First, all measurements were self-reported, meaning that the correlations observed here may be explained by the common method variance (Lindell and Whitney, 2001). Although many validated self-report questionnaires are available (Lay, 1986; Steel, 2010), the nature of procrastination should be captured using hierarchical multiple methods, such as observation from others (Rotenstein et al., 2009), laboratory tasks (Ferrari and Tice, 2000), and ecological momentary assessments (Wieland et al., 2018). Therefore, future research should use an objective indicator to measure procrastination.

Second, this study was a cross-sectional survey. Therefore, the direction of causality between procrastination and internalizing symptoms is unclear. In the case of depression, symptoms of sluggish activity might cause procrastination (Beutel et al., 2016). Regarding anxiety, previous studies have indicated that anxiety might increase procrastination (Steel, 2007). As an exception, Rozental et al. (2017) conducted a randomized clinical trial and reported that procrastination interventions reduced anxiety symptoms. Therefore, future studies should reveal the causal relationship between procrastination and internalizing symptoms using longitudinal or intervention studies.

Third, one of the six items of the ASRS Part A represents procrastination: “When you have a task that requires a lot of thought, how often do you avoid or delay getting started?” (Ustun et al., 2017) This overlap between measurement of ASRS and GPS may cause underestimation of the effect of procrastination. This measurement contamination could be avoided by utilizing the severity measure of ADHD excluding the procrastination item.

Finally, data for socioeconomic status, education level, intelligence quotient, or any other confounders were not collected. For instance, internalizing symptoms are known to be associated with the absence of partners (Roohafza et al., 2014), which may play an important role in coping with procrastination (Ferrari et al., 1999). Furthermore, the proportion of models in which procrastination explained internalizing symptoms was low, *R*^2^=0.090 for depressive symptoms and *R*^2^=0.176 for anxiety symptoms. The reason for this low explanatory rate results might be related to the attributes of the sample selected for this study. The attributes such as younger age and being a student affect procrastination (Beutel et al., 2016), which are inconsistent with the characteristics of the current participants. The present study focused on participants on the cusp of adulthood (18–30years old). The age group of 30 was the most frequently reported age group in low and high ADHD groups (17.4–18.1%). The largest age group of candidates enrolling and entering university/junior college in Japan is 18–22years. However, the proportion of 18–22years old in this sample is small: 14 (14.9% in ADHD high group) and 11 (8.3% in ADHD low group). Considering that 58.1% of students in Japan enter junior college/university (Ministry of Education, Culture Sports, Science and Technology, 2020), the proportion of students in this sample is relatively low. Since the sample’s age range in this study was different from those of the previous studies, the *R*^2^ of this model in the hierarchical multiple regression analysis might be low. Thus, confounding factors such as annual income, education, and other psychiatric disorders should be considered in future studies.

## Conclusion

This study revealed that people with higher ADHD symptom levels exhibited procrastination more frequently and showed more internalizing symptoms such as depression and anxiety. Focusing on procrastination would be helpful for those who suffer from ADHD and internalizing symptoms. However, there was no moderating effect of ADHD symptoms on the association between procrastination and internalizing symptoms. We should examine more precise and valid hypotheses and underlying mechanisms of procrastination in high and low ADHD symptom groups.

## Data Availability Statement

The raw data supporting the conclusions of this article will be made available by the authors, without undue reservation.

## Ethics Statement

The studies involving human participants were reviewed and approved by the institutional Ethics Review Committee on Research with Human Subjects of the first author’s affiliation (2018-282). Written informed consent for participation was not required for this study in accordance with the national legislation and the institutional requirements.

## Author Contributions

MO designed and conducted the survey, undertook the statistical analyses, and wrote the draft manuscript. TT, YN, and HK managed the survey and all other issues related to conducting the research. MO, TT, YN, and HK contributed to the critical revision and have approved the final version of the manuscript. All authors contributed to the article and approved the submitted version.

## Funding

This study was supported by grants from the Ibuka fund and JSPS KAKENHI grant Number 202023103.

## Conflict of Interest

The authors declare that the research was conducted in the absence of any commercial or financial relationships that could be construed as a potential conflict of interest.

## Publisher’s Note

All claims expressed in this article are solely those of the authors and do not necessarily represent those of their affiliated organizations, or those of the publisher, the editors and the reviewers. Any product that may be evaluated in this article, or claim that may be made by its manufacturer, is not guaranteed or endorsed by the publisher.

## Abbreviations

ADHD: Attention-deficit hyperactivity disorder
ASRS: Adult ADHD Self-Report Scale
ADHD-H: Higher-level ADHD symptoms group
ADHD-L: Lower-level ADHD symptoms group
AIC: Akaike information criterion
BIC: Bayesian information criterion
GPS: General Procrastination Scale
STAI-JYZ: Japanese version of the State–Trait Anxiety Inventory
PHQ-9: Patient Health Questionnaire
